# Trends and driving forces of agricultural carbon emissions: A case study of Anhui, China

**DOI:** 10.1371/journal.pone.0292523

**Published:** 2024-02-12

**Authors:** Yanwei Qi, Huailiang Liu, Jianbo Zhao, Shanzhuang Zhang, Xiaojin Zhang, Weili Zhang, Yakai Wang, Jiajun Xu, Jie Li, Yulan Ding

**Affiliations:** School of Economics & Management, Xidian University, Xi’an, China; King Fahd University of Petroleum and Minerals, SAUDI ARABIA

## Abstract

To facilitate accurate prediction and empirical research on regional agricultural carbon emissions, this paper uses the LLE-PSO-XGBoost carbon emission model, which combines the Local Linear Embedding (LLE), Particle Swarm Algorithm (PSO) and Extreme Gradient Boosting Algorithm (XGBoost), to forecast regional agricultural carbon emissions in Anhui Province under different scenarios. The results show that the regional agricultural carbon emissions in Anhui Province generally show an upward and then downward trend during 2000–2021, and the regional agricultural carbon emissions in Anhui Province in 2030 are expected to fluctuate between 11,342,100 tones and 14,445,700 tones under five different set scenarios. The projections of regional agricultural carbon emissions can play an important role in supporting the development of local regional agriculture, helping to guide the input and policy guidance of local rural low-carbon agriculture and promoting the development of rural areas towards a resource-saving and environment-friendly society.

## Introduction

That the rapid growth of greenhouse gas emissions and carbon dioxide as the main component of greenhouse gases results in ever-increasing carbon emissions and the ever-rising global temperature [1, 2]. According to the communique issued by the World Meteorological Organization, the global average annual temperature rose by 0.7°C during the century from the end of the 19th century to the end of the 20th century, so that the climate change caused by greenhouse gases has become a severe challenge for all mankind in the 21st century [3, 4]. According to the second assessment report of the Inter-government Panel on Climate Change (IPCC), among the global greenhouse gas emissions, the largest emissions come from energy use which accounts for 24%; the second is the conversion of deforestation into land of 18%; agriculture, industry and transportation industries share the third place, accounting for 14% respectively [5, 6]. The environmental problems caused by carbon emissions have gradually attracted the attention of governments all over the world. The Office of Countermeasures Coordination Group on Climate Change under the National Development and Reform Commission of China classifies the emissions sources of greenhouse gas into five categories, of which the carbon emissions from agricultural sources are the second. The carbon emissions from agricultural production activities account for 10.97% of the total carbon emissions, second only to the carbon emissions from energy consumption. Agricultural carbon emissions have become one of the main sources of carbon emissions growth [7, 8].

How to take effective measures to reduce agricultural carbon emissions has become an urgent issue. Anhui Province, located in East China, spans the Yangtze River and Huaihe River from north to south. It is abundant in agricultural resources, also serving as one of China’s traditional major agricultural provinces and one of the five major grain transport provinces [9, 10]. With the growing of global warming, agricultural disasters have broken out repeatedly in Anhui Province, which renders the agricultural production in Anhui Province face new challenges. In order to cope with the adverse impact of climate warming on the sustainable development of agriculture in Anhui Province, it is necessary to formulate countermeasures and actively carry out agricultural low-carbon emission reduction work [11, 12].

Experts and scholars have conducted a lot of research and made efforts on agricultural carbon emissions. At present, researches on agricultural carbon emissions mainly focus on the following four aspects: Firstly, calculation of agricultural carbon emissions sources. For example, it is found that agricultural fertilizer is the main source of agricultural carbon emissions [13]. It is showed that the largest single source of carbon emissions is animal excreta deposited during grazing [14]. It is found that ruminants, rice fields and biomass combustion are the main sources of agricultural carbon emissions [15]. Secondly, factors affecting agricultural carbon emissions. For instance, it is found that per capita GDP, planting structure, population density and urbanization level are the main factors affecting agricultural carbon emissions [16]. It is showed that agricultural production efficiency, agricultural structure, development level of agricultural economy, agricultural population size, urbanization, mechanization and degree of natural disasters are listed as the influencing factors of agricultural carbon emissions [17]. It is found that technological progress, agricultural infrastructure, human capital, and public investment are the factors to affect agricultural carbon emissions [18]. Thirdly, temporal and spatial changes of agricultural carbon emissions. For example, the research of the temporal and spatial change trend of greenhouse gas emissions in Anhui Province, China shows that cities in the center and the north of China had higher greenhouse gas emissions than that in the south of China [19]. It is explored that the temporal and spatial changes of the carbon footprint of cotton production in China are mainly in the northwest region, the Yellow River valley and the Yangtze River valley [20]. The research of the spatiotemporal characteristics of agricultural carbon emissions in Jiangsu Province, China is conducted from the three carbon sources of agricultural land use, rice planting and livestock breeding [21]. The research of the temporal and spatial changes of carbon footprint of grain crops in China shows that carbon emissions of rice, wheat and corn products increases slightly respectively [22]. Fourthly, the relationship between agricultural carbon emissions and economic growth. For example, the research of the decoupling relationship between carbon dioxide emissions and agricultural economic growth in 30 provinces in China from 1997 to 2014 shows that there were many periods of strong decoupling between carbon dioxide emissions and agricultural production value in East China from 1997 to 2014 [23]. The research of the relationship between agricultural carbon dioxide emissions and agricultural economic growth in Sichuan Province, China shows a linear increase in the relationship between the two [24]. The research of Iran’s carbon dioxide emissions and economic growth shows that there is a strong positive correlation between the two [25]. Fifthly, policy recommendations for agricultural carbon emission reduction. For instance, the research of the environmental improvement of agricultural carbon reduction shows that differentiated carbon reduction policies should be put in place according to regional and time differences [26]. At the same time, a carbon reduction market trading mechanism and compensation policy should be formulated to strengthen regional cooperation and form a synergistic effect on emission reduction. The research of the impact of development and utilization of water and soil resources on agricultural carbon emissions shows as follows: agricultural technology should be improved with the measures of land consolidation, scale management, water-saving irrigation and crop fallow rotation system to improve agricultural energy utilization efficiency and promote carbon emission reduction [27]. To sum up, there is an increasing number of researches on agricultural carbon emissions and ever-expanding research contents and perspectives. However, there are few demonstrative studies on regional agricultural carbon emissions, and insufficient guidance on the healthy development of regional agriculture, the control of carbon emissions and protection of agricultural ecology.

The above scholars’ research has contributed to the measurement of agricultural carbon emissions, the efficiency of carbon emissions, the relationship between agricultural carbon emissions and economic growth, and the influencing factors of agricultural carbon emissions, making the research on agricultural carbon emissions more and more abundant and in-depth. However, there is no research on the prediction of agricultural carbon emissions, especially on the prediction of regional agricultural carbon emissions using machine learning and neural networks. In this paper, we will use machine learning to construct a combined prediction model for the prediction of regional agricultural carbon emissions, and use agriculture in Anhui Province as the research object for empirical evidence.

The research question of this paper is: Is the LLE-PSO-XGBoost model constructed in this paper effective in predicting the range of agricultural carbon emissions in Anhui Province in 2030? And the prediction error performance is good?

If the future changes of the seven factors affecting agricultural carbon emissions in Anhui Province, such as the number of agricultural population, rural GDP per capita, average annual disposable income of rural residents, agricultural industrial structure, agricultural machinery power, mechanized arable land area, and agricultural energy efficiency, are inputted into the LLE-PSO-XGBoost carbon emission prediction model constructed in this paper, then a prediction of agricultural carbon emissions in Anhui Province in the 2030 range in 2030, and the prediction performance is better than the prediction performance of other comparative models.

The innovative points of this paper are as follows:

1. A combined prediction model based on machine learning is given for the prediction problem of agricultural carbon emissions, and the validity of the model is verified empirically on agricultural data in Anhui Province.
2. The machine learning prediction model proposed in this paper has better prediction accuracy on agricultural data in Anhui Province compared with other models.
3. The combined prediction model proposed in this paper is used to forecast carbon emissions from agriculture in Anhui Province for the next nine years under multiple scenarios.

The theoretical significance of this study is that it enriches the agricultural carbon emission prediction method, proposes a combined prediction model, and verifies its validity and the excellent performance of the model for agricultural carbon emission prediction.

The practical significance of this study is that the prediction of regional agricultural carbon emissions can help to play an important role in supporting the development of local regional agriculture, helping to guide the input and policy guidance of local rural low-carbon agriculture and promoting the development of a resource-saving and environment-friendly society in rural areas.

## Correlation theory

### Locally linear embedding (LLE)

Locally Linear Embedding (LLE) as a nonlinear dimensionality reduction method proposed by San T. Roweis and Lawrence K. Saul can project the feature vectors of the high-dimensional space to the low-dimensional space. While completing the dimensionality reduction of the feature vectors, the linear structure between the adjacent feature parameters before and after the calculation is basically unchanged [28, 29]. There are many ways to maintain the local structural characteristics of samples. Different maintaining methods correspond to different manifold algorithms. LLE reduces dimensions through abandoning the globally optimal dimension reduction among all samples, that is only ensuring the local optimization. At the same time, it is assumed that the sample set is placed locally to meet the linear relationship so that the computational effort of dimensionality reduction can be further reduced.

### eXtreme gradient boosting (XGBoost)

eXtreme Gradient Boosting (XGBoost) is a new integrated learning method of gradient boosting proposed by Chen Tianqi (Chen T) who has made improvements in the algorithm to achieve higher accuracy [30]. The traditional GBDT (Gradient Boosting Decision Tree) algorithm only uses the first derivative. The current value of the n^th^ tree is related to the residual of the first n-1 tree, which is difficult to achieve distribution. XGBoost applied the second-order Taylor expansion of the loss function, and adds a regular term to balance the complexity of the model and the decline of the loss function, so as to obtain the optimal solution as a whole, avoiding over fitting to some extent.

### Particle swarm optimization (PSO)

PSO, known as Particle Swarm Optimization, is an intelligent swarm optimization algorithm proposed by J. Kennedy and R.C. Eberhart et al., which is mainly used to solve optimization problems [31]. Particle Swarm Optimization (PSO) is a global random search algorithm based on swarm intelligence, which is proposed by simulating the migration and clustering behavior of birds in the process of foraging. It realizes the search of the optimal solution in complex space through the cooperation and competition among individuals on the basis of the concepts of "Swarm" and "Evolution". However, the Particle Swarm Optimization algorithm is not required to operate on individuals. Instead, it regards individuals in the swarm as particles without mass and volume in the search space. Each particle moves in the solution space at a certain velocity and moves closer to the best position in the history of the particle itself and the population, so as to realize the evolution of candidate solutions.

### LLE-PSO-XGBoost model

The research method used in this paper is the combined LLE-PSO-XGBoost prediction model, where LLE (Locally Linear Embedding) is used in this thesis to reduce the dimensionality, i.e. to reduce the dimensionality of multiple features with multicollinearity, in order to remove the mutual influence between variables; PSO (Particle Swarm Optimization (PSO) is used to find the optimal hyper-parameters, because there are various choices of hyper-parameters and different hyper-parameters have different results on the prediction accuracy performance, we need to find the optimal hyper-parameters. The model is used to forecast the future carbon emissions of Anhui Province.

The research process and steps are:

1. The original data between 2000 and 2021 are subjected to feature dimensionality reduction by LLE to get the new data after dimensionality reduction;
2. Divide the new data into training set and test set, and input the test set into XGBoost model;
3. Use PSO algorithm to tune the parameters of XGBoost model, and get the model XGBoost with optimal parameters;
4. Construct a combined LLE-PSO-XGBoost prediction model. Then the combination model is used to test the test set for regression indicators;
5. Compare the regression index with other models to prove that the combined model has the best prediction performance;
6. Finally, the combined model was used to predict the regional agricultural carbon emissions in Anhui Province between 2022 and 2030.

In this paper, Mean Square Error (MSE), mean absolute error (MAE), Root Mean Square Error (RMSE), Mean Absolute Percentage Error (MAPE) are used as the evaluation function of the combined prediction model to measure the effect of prediction model of the regional agricultural carbon emission.

## Index selection and data processing

### Index system construction

The STIRPAT model shows a great advantage in dealing with the multicollinearity of data due to its logarithmic transformation on the original data. At the same time, it can better explain the relationship between regional agricultural carbon emissions and various driving factors in demonstrative analysis. Therefore, this paper uses STIRPAT model to analyze the impact of various driving factors on regional agricultural carbon emissions in Anhui Province. The traditional IPAT model (environmental pressure model) is improved and the STIRPAT model is proposed [32].

This paper also expands the driving factors that affect the agricultural carbon emissions in Anhui Province, and studies the impact of the expanded driving factors on the regional agricultural carbon emissions in Anhui Province, including the number of agricultural population, the level of social and economic development (rural per capita GDP, average annual disposable income of rural residents, agricultural industrial structure), and the level of scientific and technological progress (agricultural machinery power, area under mechanized cultivation, agricultural energy efficiency), the logarithmic equation after expanding the driving factors can be expressed as:

lnI=m+blnP+clnA+dlnT+elnS+flnE+glnN+hlnJ+n (1)

Wherein, independent variables *S*, *E*, *N*, *J*, etc. are the driving factors of agricultural industrial structure, agricultural energy efficiency, annual disposable income of rural residents, and area under mechanized cultivation, etc.

### Data processing

Due to that different evaluation indexes tend to have different dimensions, the values vary greatly, which affects the results of data analysis. Standardization, also known as mean removal, is a necessary work for data preprocessing. In order to ensure equal importance of each feature, standardization can be performed to scale the data to a specific range. The standardized conversion equation is as follows:

$$\tilde{x}=\;\frac{x-\bar{x}}{S_{x}}$$ (2)

Where, $\bar{x}$ is mean value of explanatory variable *x*, *S*_*x*_ is the standard deviation of explanatory variable *x*, $\tilde{x}$ is the data after standardization.

## Demonstrative analysis

### Interpretation of data

Located in the southern region of China, straddling the Yangtze and Huai rivers to the north and south, Anhui Province is rich in agricultural resources and is also traditionally one of China’s major agricultural provinces and one of the top five grain transfer provinces. Rice production, in particular, ranks among the top in China’s rice production. In recent years, Anhui Province has proposed to take the path of low-carbon and green agricultural development, and a study of agricultural carbon emissions in Anhui Province has a representative role to play.

The data source used in this thesis is mainly the Anhui Statistical Yearbook, which is published annually by the Anhui Provincial Bureau of Statistics and systematically includes statistics on all aspects of the economy and society of the province and its cities and counties in the previous year, focusing on the achievements of the statistical department in serving the construction of modern and beautiful Anhui in the new stage, and is an informative yearbook comprehensively reflecting the situation of the national economy and social development of Anhui Province. The content is divided into 23 chapters and appendices, namely: General; National Economic Accounts; Population; Employed Persons and Wages; Investment in Fixed Assets; Energy Production and Consumption; Finance and Finances; Price Indexes; Urban and Rural People’s Lives; Overview of the Cities; Natural Resources and Environmental Protection; Agriculture; Industry; Construction; Transportation and Post and Telecommunication; Domestic Trade; Foreign Trade and Economy; Tourism; Education and Science and Technology; Health and Social Services; Culture and Sports; Public Administration and Others; Culture and Sports; Public Administration and Others; Provincial and County Main Economic Indicators and Ranking. The appendix part mainly includes: basic situation of non-farm employment of rural migrant workers in Anhui, e-commerce situation of enterprises and so on. In order to help readers to understand and use the relevant data, each chapter is accompanied by brief descriptions and explanations of the main indicators, introducing the scope of statistics and statistical methods.

The data related to agricultural carbon emissions and index system used in this paper are based on the Anhui Statistical Yearbook and the China Rural Statistical Yearbook from 2000 to 2022, and the historical data are all converted into 2005 constant prices according to the deflator. When calculating the data related to agricultural carbon emissions in Anhui Province, this paper applied the coefficient reference data of IPCC. When training the LLE-PSO-XGBoost combined model, the data of the carbon emission indicator system from 2000 to 2018 will be used as the training set, and the data from 2019 to 2021 will be used as the test set. Moreover, the data will be standardized before the training of the combined model, and the anti-standardization operation will be performed after the completion of the training, which will be regarded as the final output result. Statistical characteristics of the original data are shown in Table 1:

**Table 1:** Statistical characteristics of the original data.

Variable	Min	Max	Mean	Std
*I*	1137.603	1480.417	1341.731	100.149
*P*	0.315	0.465	0.384	0.053
*A*	0.134	0.888	0.433	0.243
*T*	0.298	0.692	0.526	0.135
*S*	0.445	0.554	0.500	0.028
*E*	0.040	0.112	0.070	0.022
*J*	0.359	0.767	0.479	0.158
*N*	0.193	1.837	0.750	0.534

**Fig 1:**
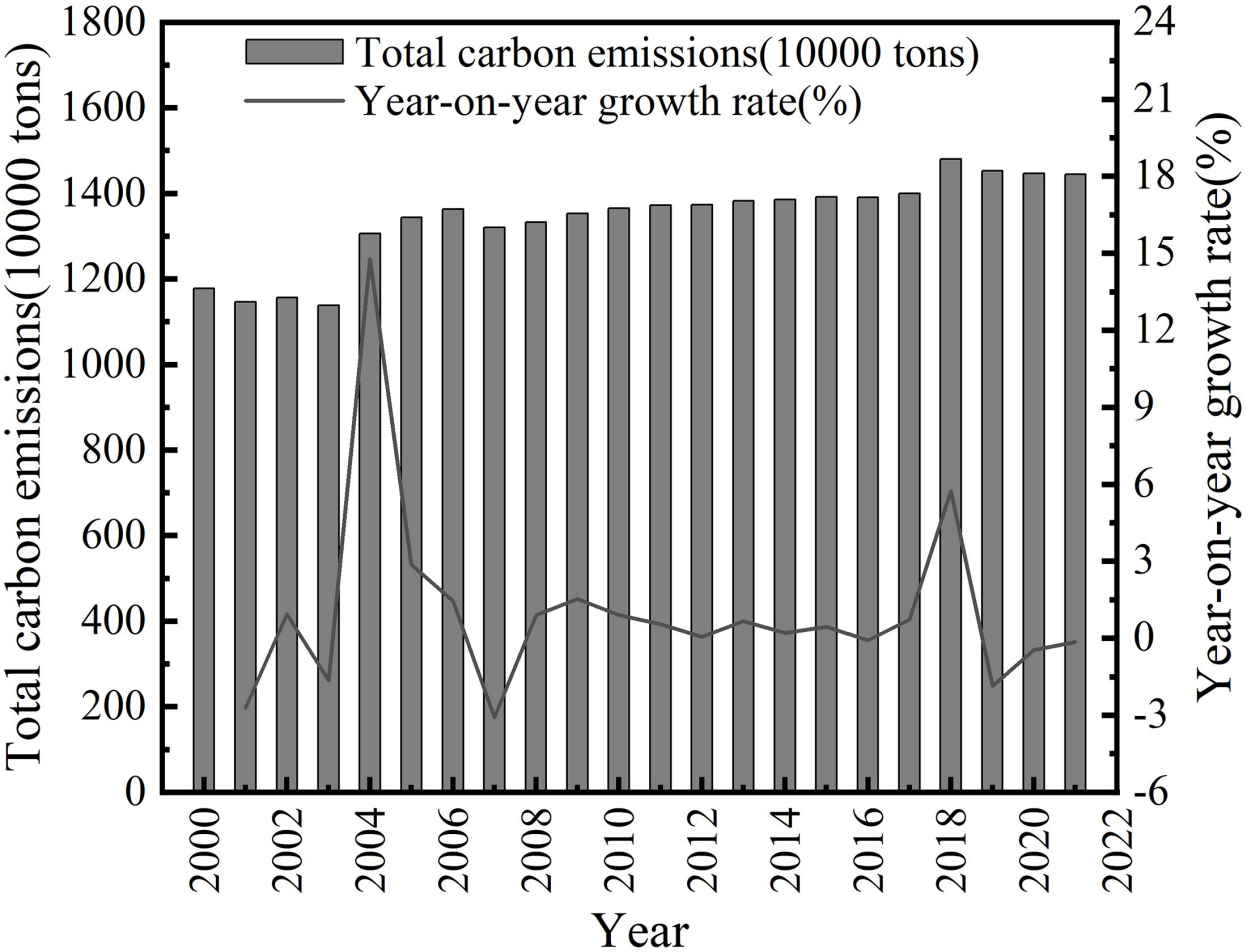
Historical agricultural carbon emission and growth rate in Anhui province.

The agricultural carbon emissions and growth rates of Anhui Province for each year are shown in Fig 1. It can be seen that the total amount of agricultural carbon emissions in Anhui Province shows a gentle upward trend, increasing from 1177.84 tons in 2000 to 1144.01 tons in 2021; while the annual growth rate of agricultural carbon emissions in Anhui Province shows a trend of first increasing and then decreasing, from -2.70% in 2001 to 14.78% to -0.15% in 2021.

**Fig 2:**
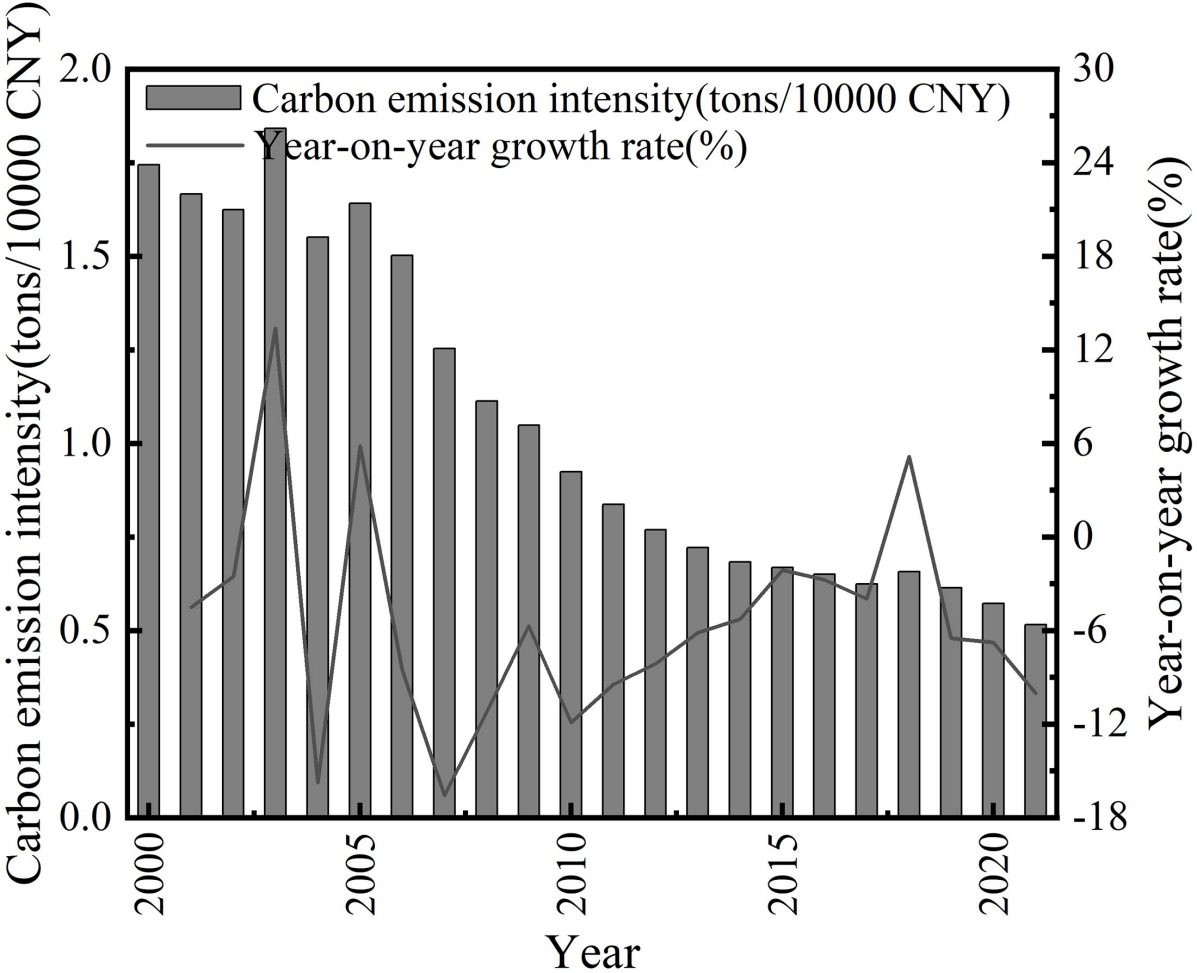
Historical agricultural carbon emission intensity and growth rate in Anhui province.

The agricultural carbon emission intensity and growth rate of Anhui Province for each year are shown in Fig 2. The growth rate of agricultural carbon emission intensity in Anhui Province in each year shows a steady and unchanging trend, from -4.50% in 2001 to -10.03% in 2021.

### Multicollinearity test

Multicollinearity refers to the correlation between explanatory variables in the model, and multicollinearity is a common problem in multiple regression models. Multiple collinearities among explanatory variables can be tested by the variance inflation factor (VIF) [33].

### LLE feature dimensionality reduction

Due to the severe multicollinearity among various indexes, this paper uses the (LLE) Feature Dimensionality Reduction algorithm to reduce the dimensionality of the index system of the original data (agricultural population, rural per capita GDP, annual disposable income of rural residents, agricultural industrial structure, agricultural machinery power, area under mechanized cultivation, agricultural energy efficiency) to obtain the new data after dimensionality reduction.

### PSO-XGBoost model prediction

#### Model prediction

In this paper, the original data is analyzed by multicollinearity, and the new data is constructed by feature dimensionality reduction by LLE algorithm. The data from 2000 to 2018 will be used as the test set, and the data from 2019 to 2021 as the training set after the standardization of all the data. At first, data of the training set is trained by substituting them into XGBoost model, and hyper-parameters of the model are optimized by PSO algorithm. The PSO algorithm randomly combines the parameters within a given range, and evaluates the model effect of each parameter combination under the training set data, so as to discover a set of optimal hyper-parameters. The optimal parameter structure is obtained: *n*_*estimators*: 80, *max*_*depth*: 2, *learning*_*rate*: 0.3, generating a minimum value of the evaluation function of the regression model. At this time, the root mean square error (RMSE) of the LLE-PSO-XGBoost model is the minimum 0.0031.

**Fig 3:**
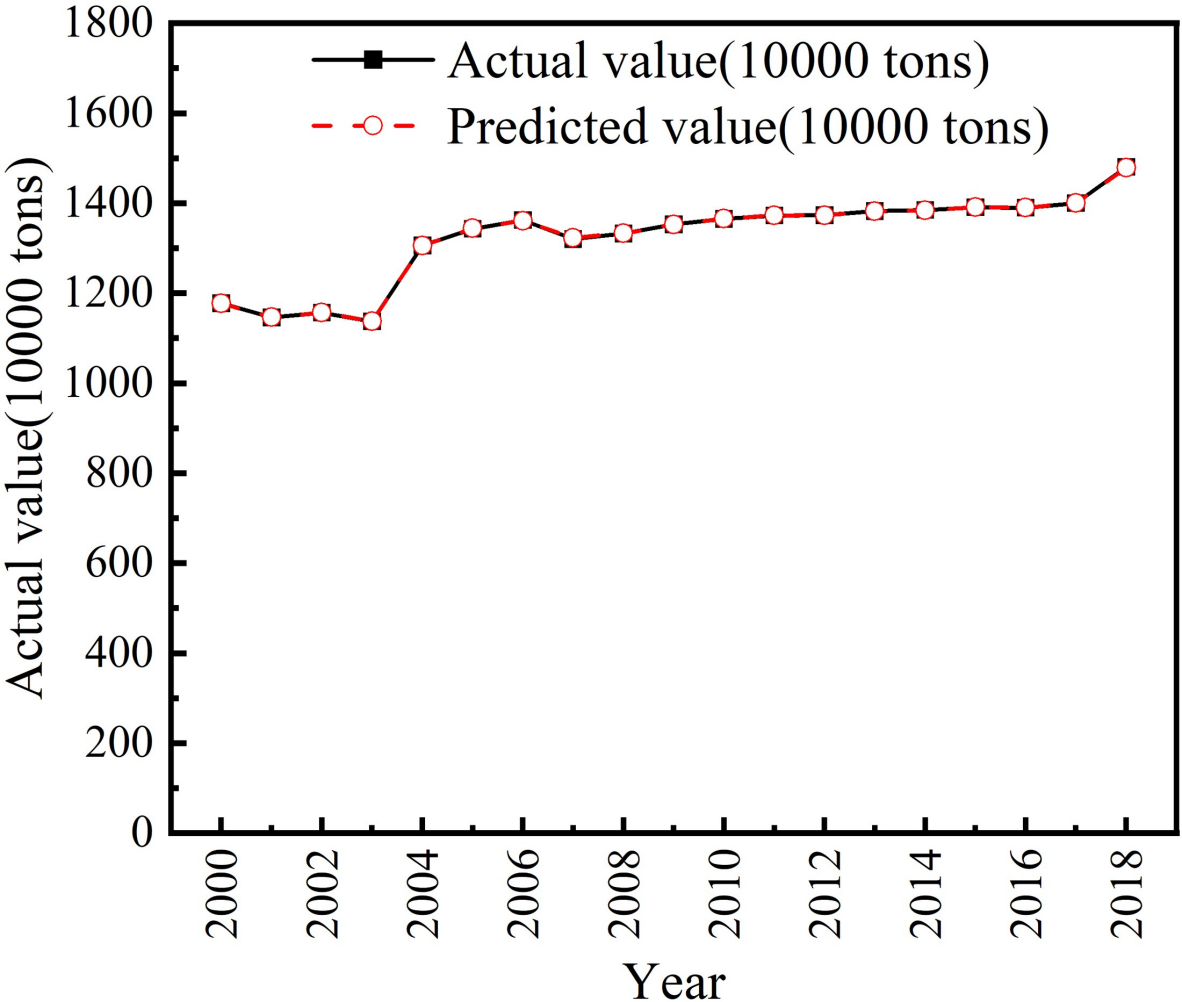
Fitting results of training set.

Fig 3 is a broken line graph of the fitting results for the training set, which can be seen that the LLE-PSO-XGBoost model shows a good fitting effect for the training set.

Then the trained LLE-PSO-XGBoost model is used to predict the data of the test set. The average prediction error rate of the test set is 0.003, which indicates that the model shows good prediction ability. In order to verify the model effect of LLE-PSO-XGBoost, it is compared with PLS-PSO-XGBoost model that using Partial Least Squares (PLS) for feature dimensionality reduction and Particle Swarm Optimization (PSO) for parameter optimization as well as PCA-PSO-XGBoost model that using Principal Component Analysis (PCA) for dimensionality reduction and Particle Swarm Optimization (PSO) for parameter optimization. The LLE-PSO-XGBoost prediction model shows the best fitting effect, the lowest average prediction error rate of 0.009.

In order to verify the model effect of LLE-PSO-XGBoost, it is compared with more machine learning and neural network models, including Support Vector Regression (SVR) and Random Forest (RF) in machine learning as well as Multilayer Perceptron (MLP) in neural network. These models use Partial Least Squares (PLS), Principal Component Analysis (PCA), Locally Linear Embedding (LLE) for feature dimensionality reduction, and Particle Swarm Optimization (PSO) for hyper-parametric optimization. The measurement indexes use evaluation functions of MSE, MAE, RMSE, MAPE, etc. The LLE-PSO-XGBoost prediction model shows the lowest evaluation function value and the best accuracy of 99%.

## Discussion

Scenario Analysis is a major method, commonly used to predict the process of possible events and explore the uncertain future. This paper applies the scenario analysis method to predict the agricultural carbon emissions of Anhui Province in 2022–2030, and sets the following five scenarios: high growth rate, average growth rate, low growth rate, positive minimum growth rate, negative maximum growth rate. The index system generates the predicted index value of 2022–2030 based on the growth rate set in these five scenarios, and then places the predicted index value into the LLE-PSO-XGBoost model, so as to predict the regional agricultural carbon emissions of Anhui Province in 2022–2030.

### Scenario analysis and parameter setting

According to the five different scenarios set above, the paper uses the different growth rates corresponding to the five scenarios to generate the predicted values of each index in the index system (seven indexes including agricultural industrial structure, agricultural machinery power, and area under mechanized cultivation) in 2022–2030.

### Prediction of agricultural carbon emissions in Anhui province

**Fig 4:**
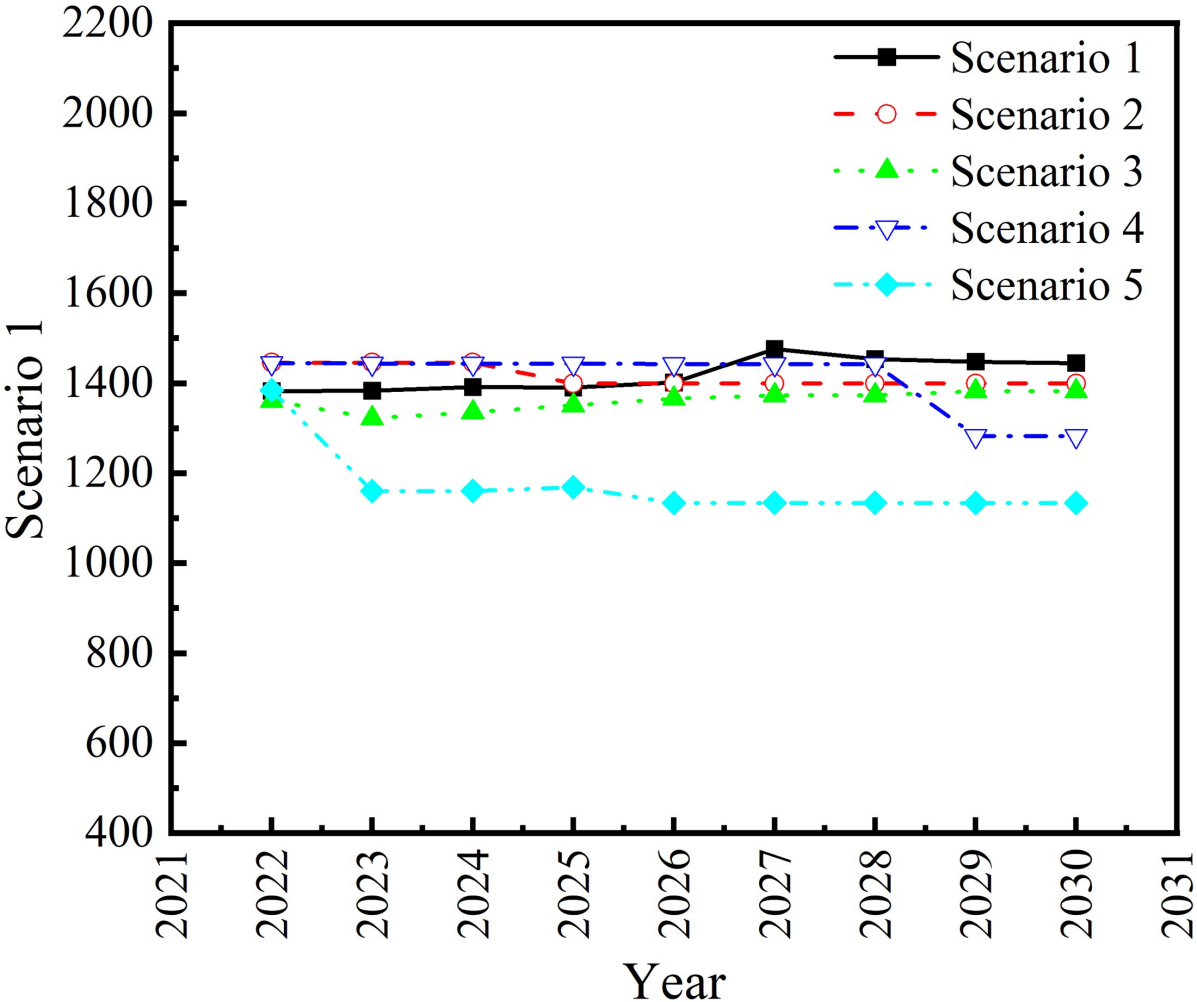
Prediction of agricultural carbon emissions in Anhui province from 2022 to 2030.

The LLE-PSO-XGBoost model is applied to predict the agricultural carbon emissions of Anhui Province under the five scenarios. The prediction results from 2022 to 2030 are shown in Fig 4.

It can be seen from Fig 4 that the predicted agricultural carbon emissions in Anhui Province in Scenario 1 shows a slow rising trend, from 13,824,200 tons in 2022 to 14,445,700 tons in 2030, an increase of 4.50% in 9 years, with an average annual growth rate of 0.56%. In Scenario 2, the predicted agricultural carbon emissions in Anhui Province shows a slow fall trend, from 14,458,100 tons in 2022 to 13,995,700 tons in 2030, a decline of 3.20% in 9 years, and an average annual decline rate of 0.40%. In Scenario 3, the agricultural carbon emissions in Anhui Province are predicted to rise slightly, from 13,608,800 tons in 2021 to 13,804,200 tons in 2030, with an increase of 1.58% in 9 years and an average annual growth rate of 0.20%. In Scenario 4, the agricultural carbon emissions of Anhui Province are predicted to decrease, from 14,448,200 tons in 2022 to 12,826,800 tons in 2030, with a decline of 11.22% in 9 years and an annual average decline rate of 1.41%. In Scenario 5, the predicted agricultural carbon emissions in Anhui Province shows a rapid fall trend, from 13,845,900 tons in 2022 to 11,342,100 tons in 2030, a decline of 18.08% in 9 years, with an average annual decline rate of 2.30%. To sum up the prediction results of the six scenarios set above, the regional agricultural carbon emissions of Anhui Province in 2030 are expected to fluctuate between 11,342,100 tons and 14,445,700 tons.

In order to maintain a low level of agricultural carbon emissions in Anhui Province in the future, regional agriculture should be strived to develop in accordance with scenarios 4 and 5, that is, to develop regional agriculture under the condition of maintaining seven indexes such as the agricultural industrial structure, agricultural machinery power, and the area under mechanized cultivation in a negative maximum growth rate or low growth rate, so as to effectively control the level of agricultural carbon emissions in Anhui Province, and promote the green and healthy development of agriculture in Anhui Province.

The contribution of this paper is to construct a combined prediction model based on machine learning, and use the model to effectively predict agricultural carbon emissions in Anhui Province, while the factors influencing agricultural carbon emissions are selected from three aspects: population, economic development and technological progress. Agricultural economic development is the most important factor in the growth of carbon emissions, and agricultural production efficiency, agricultural production structure and labor force size play an important role in curbing the growth of agricultural carbon emissions, among which agricultural production efficiency plays the largest role and has the most obvious curbing effect, while agricultural production structure and agricultural labor force size also achieve a certain curbing effect, but the effect is very small.

## Conclusion

This paper constructs a combined LLE-PSO-XGBoost carbon emission prediction model combining local linear embedding (LLE), particle swarm algorithm (PSO), and extreme gradient boosting algorithm (XGBoost), and selects seven characteristic variables, with The LLE-PSO-XGBoost combined prediction model was trained using agricultural data from Anhui Province, and after obtaining the optimal model, the prediction of agricultural carbon emissions in Anhui Province for the next nine years was carried out under five scenarios. The results show that the selected characteristics variables, such as the number of agricultural population, agricultural industry structure, per capita disposable income of rural residents, per capita agricultural GDP, agricultural machinery power representation, area of mechanically cultivated land in that year, and energy efficiency of agricultural production, can be input into the LLE-PSO-XGBoost combined prediction model to effectively predict the future agricultural carbon emissions in Anhui Province. Moreover, the prediction error rate of the combined LLE-PSO-XGBoost prediction model is 0.009, which is lower than the error rate of the PLS-PSO-XGBoost model using least squares method of 0.063, and also lower than the error rate of the PCA-PSO-XGBoost model using principal component analysis of 0.046. In addition, comparison with more machine learning models including support vector machines (KNN), random forests (RF), and multilayer perceptron (MLP), also had the best prediction performance of the individual models. Furthermore, in the five scenarios, this paper predicts the agricultural carbon emissions in Anhui Province for the next nine years, and the regional agricultural carbon emissions in Anhui Province in 2030 are expected to fluctuate between 11,342,100 tones and 14,445,700 tones. To maintain agricultural carbon emissions in Anhui Province at a low level, it should try to develop according to scenarios such as Scenario 4 and Scenario 5, i.e. develop regional agriculture while maintaining an average or lower growth rate of seven indicators such as agricultural GDP per capita, agricultural industry structure, and the level of total agricultural machinery power, to ensure healthy, environmentally friendly and sustainable development of agriculture and effectively control the level of agricultural carbon emissions in Anhui Province.

Anhui government should step up publicity to enhance awareness of the low-carbon economy in rural areas and integrate the requirements of low-carbon development into all aspects of agricultural development. They should increase investment and policy guidance in taxation, finance and other policies for low-carbon agriculture in rural areas, and increase the promotion and guidance of the low-carbon agricultural technologies developed to promote rural development towards a resource-saving and environment-friendly society.

In this paper, we have studied various factors influencing regional agricultural carbon emissions and quantitatively analyzed the trends of agricultural carbon emissions in Anhui Province, but as the region is limited to Anhui Province, there is insufficient research on other regions. The future research direction will try to investigate the factors influencing agricultural carbon emissions, carbon emission projections and policy implications in other regions. The practical significance of this thesis is to establish the LLE-PSO-XGBoost carbon emission empirical prediction model, and to carry out empirical evidence using Anhui Province agriculture as an example, hoping to serve as a reference for agricultural carbon emission research in other countries and regions.
